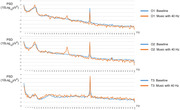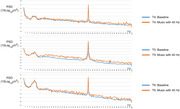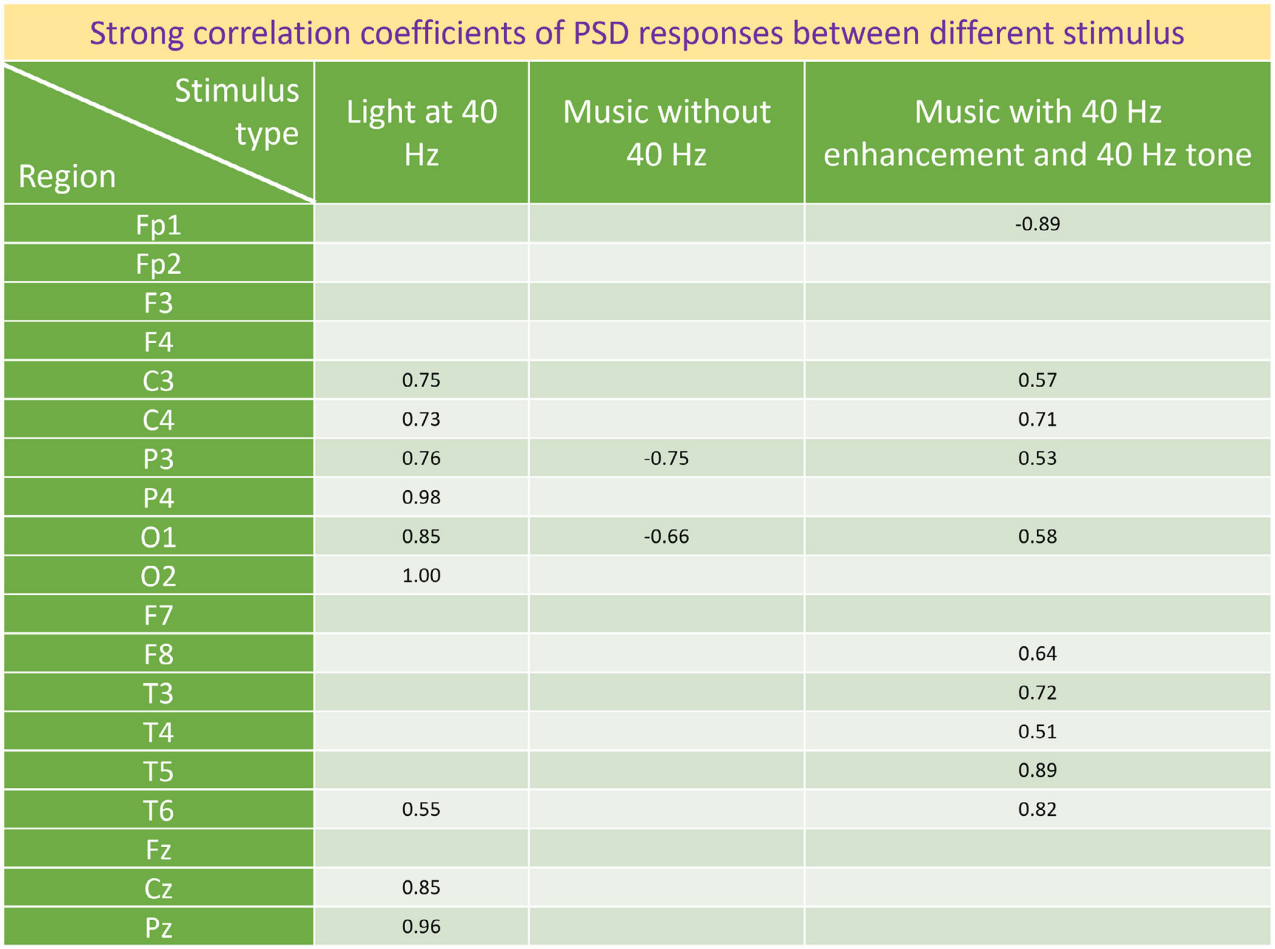# Effect of Music Stimulation on Network Activity of Patients with Dementia with Lewy Bodies

**DOI:** 10.1002/alz.089781

**Published:** 2025-01-03

**Authors:** Vishnu Shandilya Mungamuru Chenchu, Kwaku Addo‐Osafo, Joshua Stepter, Karen Elrayes, Aisha Mohammed, Sydney Kilgore, Katy Figueroa, Keith Vossel

**Affiliations:** ^1^ University of California, Los Angeles, CA USA

## Abstract

**Background:**

Dementia exhibits abnormal network activity, including altered gamma frequency (30‐100 Hz) in Alzheimer’s disease (AD). A non‐pharmacological, non‐invasive approach to AD treatment involves stimulating sensory inputs using gamma band, with 40 Hz as the most effective in eliciting a robust EEG response. Light and sound stimulation at 40 Hz reduces AD pathology in mouse models and improves cognition in humans with AD. However, the effects of this stimulation in patients with dementia with Lewy bodies (DLB) and mild cognitive impairment (MCI) is unknown, as well as whether musical sounds can be more efficient than sounds without music.

**Method:**

Individuals with DLB, MCI, AD, and controls participated in a music‐based brain stimulation study. Participants listened to music enhanced at 40 Hz with an overlapped 40‐Hz tone using headphones while connected to an EEG with 200‐to‐2000‐Hz sampling rate and 10‐20 placement. 40‐Hz flickering light was stimulated transiently at time intervals alone or in combination with sound stimulation. Participants underwent a 10‐minute initial assessment consisting of combinations of light/sound inputs followed by 1 hour of music and intermittent light stimulation. Power spectral density (PSD) and correlations of PSDs with different stimuli were measured.

**Result:**

In DLB (female, mid‐50 s), sampled at 200 Hz, PSD showed a broad increase in response during gamma frequency stimulations. Light at 40 Hz (L40) resulted in increased response at 40 Hz at O1 and O2 electrodes, with a visible spike on the PSD. Music with 40‐Hz enhancement and 40‐Hz overlaid tone (M40) resulted in a strong positive correlation (SPC; Pearson correlation coefficient ≥ 0.5) at C3, C4, P3, O1, F8, T3, T4, T5, T6 and strong negative correlation (SNC; coefficient ≤ ‐0.5) at Fp1. Previously, 4 patients (1‐AD,1‐MCI,2‐controls) were sampled at 2000 Hz, which generated very low signal‐to‐noise ratio (SNR) and responses to stimulations were variable in these groups.

**Conclusion:**

In DLB, music with 40 Hz enhancement and tone increased gamma frequency response, especially in temporal regions, with strong positive correlations. Ongoing studies will determine the responses of brain oscillations to music‐based stimulations in additional participants with DLB, MCI, AD, and controls.